# Comparison between the American and the European Systems of Monitoring Adverse Effects of Dietary Supplements and Their Usefulness on the Polish Market

**DOI:** 10.3390/ijerph20020902

**Published:** 2023-01-04

**Authors:** Kacper Wróbel, Beata Zastawna, Anna Justyna Milewska, Michał Marczak, Remigiusz Kozłowski

**Affiliations:** 1 Department of Management and Logistics in Healthcare, Medical University of Lodz, 90-131 Lodz, Poland; 2 Department of Biostatistics and Medical Informatics, Medical University of Bialystok, 15-089 Bialystok, Poland; 3 Centre for Security Technologies in Logistics, Faculty of Management, University of Lodz, 90-237 Lodz, Poland

**Keywords:** dietary supplements, side effects, CAERS, EudraVigilance, Nutrivigilance

## Abstract

Many Polish patients do not inform physicians about supplements they use in addition to prescribed medicines. This may be because they consider dietary supplements as being rather natural products that cannot cause health problems. Although dietary supplements may produce side effects, Poland’s food safety system and medical statistics do not recognise the necessity of reporting such cases. However, a different approach is observed in France and the United States where adverse effects of food supplements as well as drugs are reported. The aim of this study was to determine the need for creating in Poland a general model of a register monitoring dietary supplements and their adverse effects. In order to achieve this goal, a detailed comparison between the American and European monitoring systems was made. It showed the relationship between negative symptoms caused by specific components in supplements and t profiles of patients who reported side effects. Additionally, it was found that there is a real risk associated with side effects caused by dietary supplements. Therefore, it necessary to establish in Poland a special system for recording such cases as it should be beneficial to patients, including polypragmatic patients.

## 1. Introduction

For decades, dietary supplements have been used by consumers from various countries. Dietary supplements by their definition are a concentrated source of vitamins, minerals, and/or other physiologically active ingredients which are supposed to supplement the regular diets of individuals [1]. On the American market, their increased popularity was noted as early as in the 1970’s [2]. Moreover, it has been known for years that consuming dietary supplements may be associated with the occurrence of adverse effects [3], with the underlying causes differing and ranging from cases of incorrect use or misuse of a dietary supplement [4,5], to unwitting consumption of products contaminated with various substances [6].

Despite the fact that research studies conducted in the United States show that safety of consumption of dietary supplements, including their adverse effects, is a topic that patients often bring up during their appointments with physicians [7], studies conducted in Japan, on the other hand, show that most patients do not inform their doctors or pharmacists about consumed dietary supplements, assuming that they are completely safe and neutral in the context of the concurrent pharmacotherapy [8]. Interestingly, compared to other nationalities, Poles seem to be more often aware that using dietary supplements may have an impact on the results of medical laboratory tests and consider it proper to inform lab personnel about their consumed products [9]. Unfortunately, however, still almost one-third of patients who participated in a survey carried out in one of Polish oncology centres indicated that they had not informed their physicians about supplements they were using concurrently [10]. This approach may result from a growing belief among Poles that supplements are not medicines or pharmaceuticals but rather natural products that enhance a regular healthy diet. This phenomenon may be reinforced by media marketing ads for medicines, which point out that the advertised product is a medicine, not a supplement [11].

Even if an adverse effect is linked with a dietary supplement consumed by a patient, then considering the Polish model of healthcare, neither a doctor nor patients themselves have the means and responsibility to report, code, and notify on the adverse reaction to a certain product [12]. The International Statistical Classification of Diseases and Related Health Problems (ICD-10) does not propose any codes for adverse effects of substances that are neither typical foods nor medicines, i.e., dietary supplements. Dietary supplements are not explicitly mentioned in the section devoted to “External causes of morbidity and mortality.” Moreover, they do not meet the criteria that would make it possible to include them in the categories listed under “Sequelae of injuries, of poisoning and of other consequences of external causes (T90–T98)” [13]. Thematically the closest are the following items: “T96 Sequelae of poisoning by drugs, medicaments and biological substances”; sequelae of poisoning classified in categories T36–T50; “T97 Sequelae of toxic effects of substances chiefly nonmedicinal as to source”; and sequelae of toxic effects classified in categories T51–T65. It is rare, however, that the consumption of supplements does indeed cause toxic or acute effects, recognised as poisoning [14]. Less pronounced symptoms may be coded under “Drugs, medicaments and biological substances causing adverse effects in therapeutic use (Y40–Y59)”. This section, however, is annotated with the following information: “For list of specific drugs classified under the fourth-character subdivisions, see Table of drugs and chemicals in Alphabetical Index”, which does not allow one to report adverse effects of products not included in the Index.

Hence, adverse effects of supplements are usually reported by doctors as symptomatic diagnoses, which does not allow one to estimate the frequency of events connected with particular substances or preparations [15,16]. These are, for example, skin lesions (which may be coded as diagnoses under L20, L272, L27,9, L50.1, L50.8, L50.9, or L56), digestive symptoms (codes such as, e.g., R10-R14, K30, K52, or K58), headache (R51), kidney stones (N20), etc.

Limited knowledge—both the patients’ and the doctors’—in the area of possible negative reactions to ingredients of dietary supplements [17] and the lack of methods for their reporting, results in there being no available statistics that would allow for debunking the myths surrounding the safety of these substances [18].

In the case of individuals who use many different supplements with similar compositions convinced that their larger amounts will translate into an increased health effect, adverse reactions may cumulate, making it difficult to ascertain whether the large doses, the interactions, or one particular substance is responsible for the ailments (ICD 10: “F55 Abuse of non-dependence-producing substances”) [3].

Different countries employ different methods for the notification, processing, and reporting of data concerning adverse effects of dietary supplements [19,20], but also those caused by medicines [21], including plant-based and traditional remedies [22]. The latter group in particular often consists of those borderline products that can be qualified as dietary supplements or medicines [23]. Depending on circumstances, these “borderline” substances can be treated as additives which enrich the diet of healthy individuals and potentially cure sick people [24].

The result of an analysis of individual cases of adverse effects following the consumption of a dietary supplement may as a rule be treated as an indication whether a given product may be suspected of causing the adverse health effect in question. For this reason, systemic collection of information concerning recurrent cases is of such importance, as this approach may lead to the establishment of a connection between a given product and the adverse effect; it may also allow determination of the maximum safe doses [25].

The above assumption led to the creation of a register of adverse effects that is a component of the system of the food safety assurance system. In France it is called Nutrivigilance. The purpose of the system is to promptly identify any possible negative effects connected with the consumption of certain food products and hence increase consumer safety. More precisely, the system helps in directing some specific, food safety-related topics to experts, who can perform targeted risk analysis [26]. The register, however, is not publicly available in the form of aggregated data. Based on the conclusions from analyses and notifications, the French Agency for Food, Environmental and Occupational Health and Safety (ANSES) publishes risk assessment reports. So far, published reports have investigated, among others, dietary supplements containing melatonin [27], spirulina [28], fermented red rice [29], and p-synephrine [30], as well as dietary supplements for pregnant women [31] and sportsmen [32], and energy drinks [33].

In the USA, a similar role is performed by the Center for Food Safety and Applied Nutrition (CFSAN) and the Adverse Event Reporting System (CAERS). CAERS is a database that contains information concerning adverse events and complaints about products reported to the Food and Drug Administration (FDA) in relation to food, dietary supplements, and cosmetics [34]; unlike the French register, however, the American model is available in the form of a table containing the original unprocessed data from each notification.

As far as medicines are concerned, a system called EudraVigilance operates in the European Union, dedicated to the management and analysis of information concerning suspected adverse effects of medicines that have been admitted to trading or are being tested in clinical trials in the European Economic Area (EEA). The system is operated by the European Medicines Agency (EMA) [35].

Despite the fact that dietary supplements permeate both the food and the pharmaceutical markets, especially those that due to the ingredients used may be regarded as “borderline products”, no system for the reporting of adverse effects potentially caused by the group of products in question exists in Poland.

In contrast, in Poland and France, companies placing dietary supplements on the market should notify the local food safety authority [36], while in the USA, a notification procedure is not established for regular products [24]. Regardless of this difference, it should be noted that in all countries of which ADEs reporting systems were included in this study, food companies do not need to obtain authorisation for dietary supplement marketing.

Considering the fact that the market of dietary supplements in Poland is exceptionally large, which may have an impact on the number and intensity of adverse effects—based on data sourced from three different registers (CAERS, French Nutrivigilance system database, and the EudraVigilance database) dedicated to the collection of data about adverse effects caused by foods, including dietary supplements, as well as medicines containing, e.g., ingredients common in dietary supplements—the authors undertook the study for the following reasons:

(1) to determine the needs for creating, in the future, a national register of adverse effects caused by dietary supplements by determining the degree of the presence of borderline-type supplements on the Polish market, including their potential for causing adverse effects;
(2) to prepare a general concept of a register of adverse effects for Poland, considering the various conclusions drawn from European and American experiences in the area of the functioning of such registers.

## 2. Materials and Methods

### 2.1. The French Register

The analysis was based on data sourced from the list of resolutions and opinions issued by The French Agency for Food, Environmental and Occupational Health and Safety (ANSES). Notifications concerning adverse effects caused by dietary supplements consumed in France were selected using the search engine for documents from the period 2000–2022 available on the ANSES website.

In the first phase, a list of reports concerning human nutrition was obtained. A total of 162 of these contained the word “supplement” in the title. Eventually, 24 documents issued between 2007 and 2021 were selected (Table 1), i.e., those whose title suggested that the opinion/report was issued as a result of a notification made following the consumption of a particular dietary supplement or a particular ingredient. At the same time, reports whose title suggested that they were issued on ANSES’s own initiative were excluded. Then the contents of the selected reports were analysed and the main ingredient suspected of causing the adverse effect was determined.

### 2.2. Recognising Selected Ingredients as Borderline

In order to determine a dietary supplement as “borderline”, the ingredients selected above were searched in the European database of adverse reactions to medicines, i.e., EudraVigilance. Searching the database by ingredient name led to finding seven ingredients present in both registers, i.e., the French Nutrivigilance and EudraVigilance, namely: melatonin, liquorice, green tea, glucosamine, chondroitin, echinacea, and cimicifuga. Detailed data sourced from the EduraVigilance register concerning all the aforementioned ingredients were collected for the study, considering the following: number of notifications, their distribution in respective age groups, division by sex, and the category of the entity that made the notification.

### 2.3. Borderline Products in the American Register

Similar data concerning the nature of adverse effects and patients’ profiles to that sourced from the EudraVigilance register was searched in the American CAERS database. For this purpose, first, a list of cases reported in the period 2004–2021 was filtered. Data from the register of adverse effects kept by FDA were narrowed down only to cases categorised as category 54, i.e., “Vit/Min/Prot/Unconv Diet(Human/Animal)”. Then only those notifications were selected for which the product name made it possible to categorise them as one of the seven ingredients mentioned above. The “product name” search terms for the individual ingredients are shown in the “key word” section, in Table 2, Table 3, Table 4, Table 5, Table 6, Table 7 and Table 8. In order to make it the possible to compare data from both registers, data concerning the age of consumers from the American register was converted into age brackets, in the manner mirroring the EudraVigilance register: Not Specified, 0–1 Month, 2 Months–2 Years, 3–11 Years, 12–17 Years, 18–64 Years, 65–85 Years, and More than 85 Years. A verbal description of each of the reported adverse effect, or diagnosis, was placed in one of the 27 general categories as used in the EU register, in order to enable their subsequent comparison. Those descriptions of adverse effects that were not precise enough to place them in one of the aforementioned 27 categories specified in register kept by EMA were denoted in the results as “excluded” from further analysis.

### 2.4. Assessment of Dietary Supplements Containing Borderline Ingredients

In order to assess the extent of borderline-type ingredients in dietary supplements registered on the Polish market, data concerning the number of products containing the studied ingredient submitted to the national register of dietary supplements and functional foods in the period 2007–2022 was used. The register was searched by “key words” in the register’s “ingredients” field. The key words were chosen separately for each ingredient.

For the American market, a similar assessment was performed based on searches of The Dietary Supplement Label Database (DSLD). Data in the database originates from notifications made in national population studies and voluntary notifications made by manufacturers and vendors of dietary supplements. The database covers information concerning the labels of products available on the market as well as those withdrawn, in order to ensure a comprehensive set of labels. The list is regularly updated to add new products and reflect the formulae and other changes in labels of existing products.

The key words that both the aforementioned databases were searched by are indicated in Table 2, Table 3, Table 4, Table 5, Table 6, Table 7 and Table 8.

### 2.5. Data Comparison

**Figure 1:**
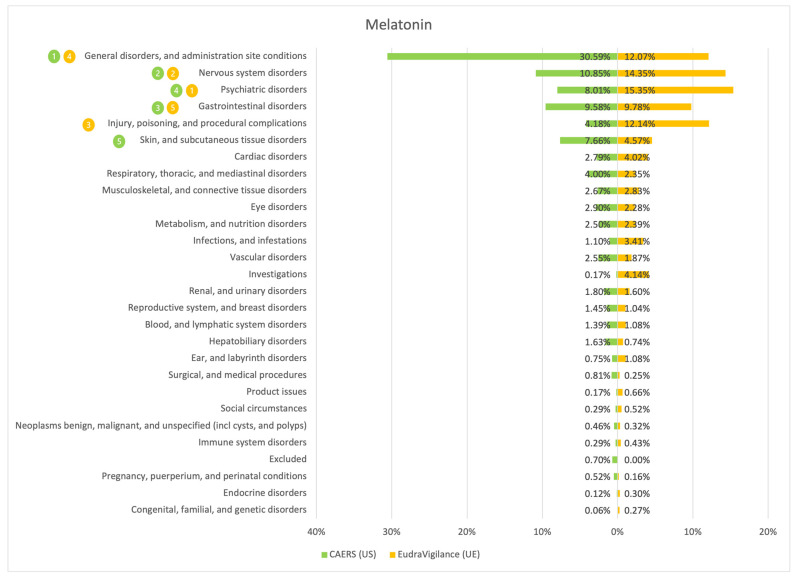
Summary of the frequency of reporting side effects of Melatonin in the US and EU registers. Data marked in green refer to the CAERS (US) database and yellow to the EudraVigilance (UE) database. Numbers 1–5 present the most commonly reported categories of adverse effects in descending order.

**Figure 2:**
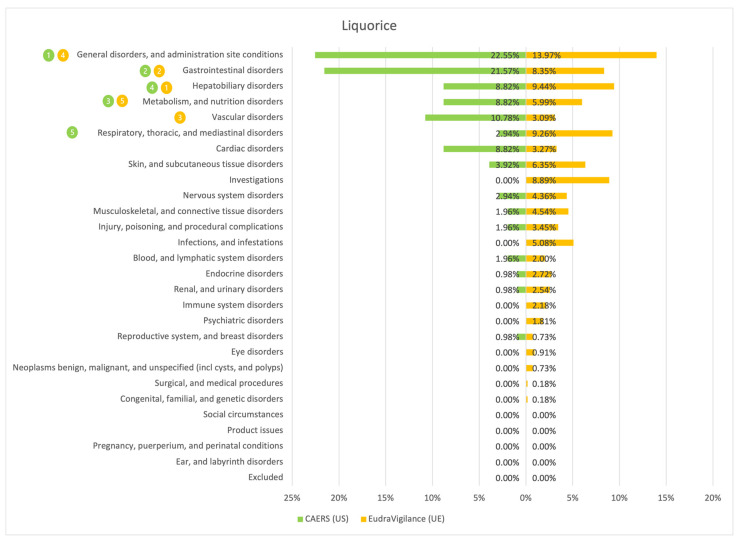
Summary of the frequency of reporting side effects of Liquorice in the US and EU registers. Data marked in green refer to the CAERS (US) database and yellow to the EudraVigilance (UE) database. Numbers 1–5 present the most commonly reported categories of adverse effects in descending order.

**Figure 3:**
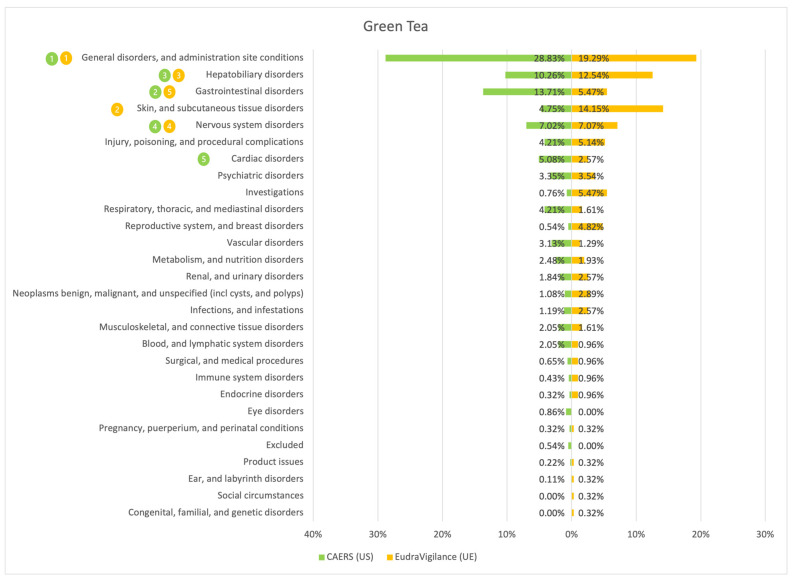
Summary of the frequency of reporting side effects of Green Tea in the US and EU registers. Data marked in green refer to the CAERS (US) database and yellow to the EudraVigilance (UE) database. Numbers 1–5 present the most commonly reported categories of adverse effects in descending order.

**Figure 4:**
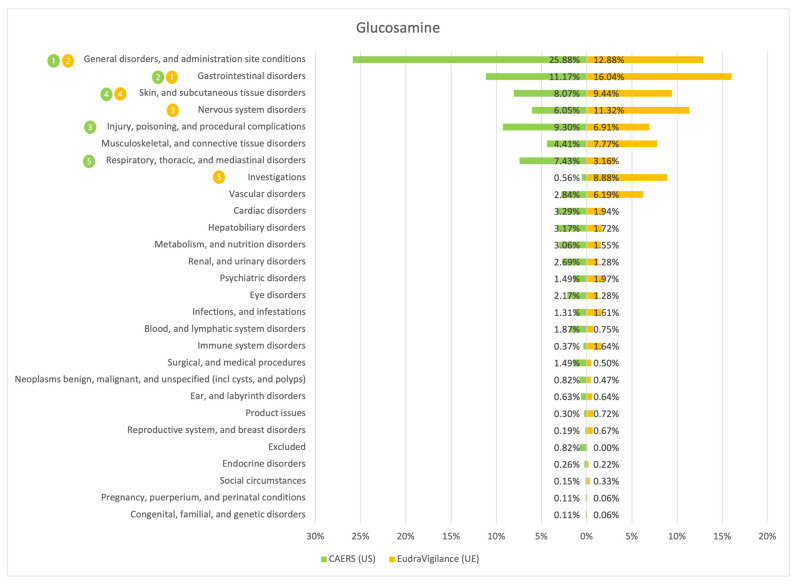
Summary of the frequency of reporting side effects of Glucosamine in the US and EU registers. Data marked in green refer to the CAERS (US) database and yellow to the EudraVigilance (UE) database. Numbers 1–5 present the most commonly reported categories of adverse effects in descending order.

**Figure 5:**
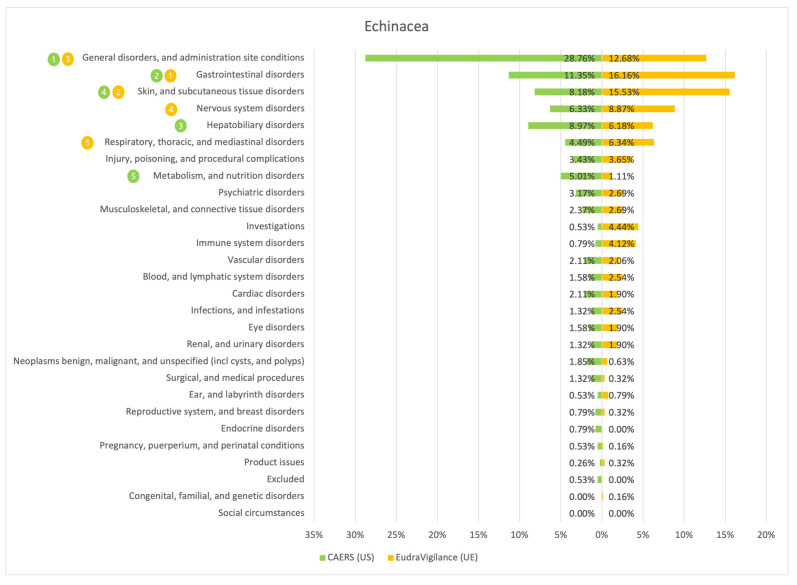
Summary of the frequency of reporting side effects of Echinacea in the US and EU registers. Data marked in green refer to the CAERS (US) database and yellow to the EudraVigilance (UE) database. Numbers 1–5 present the most commonly reported categories of adverse effects in descending order.

**Figure 6:**
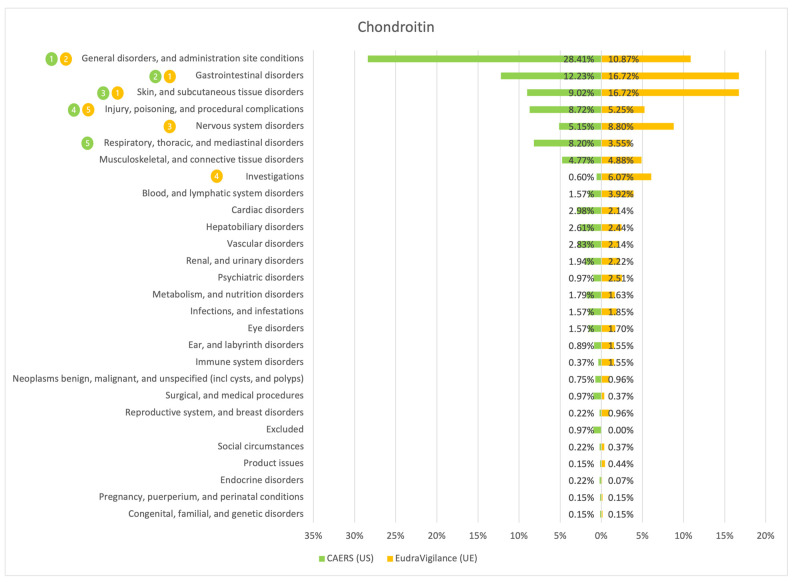
Summary of the frequency of reporting side effects of Chondroitin in the US and EU registers. Data marked in green refer to the CAERS (US) database and yellow to the EudraVigilance (UE) database. Numbers 1–5 present the most commonly reported categories of adverse effects in descending order.

**Figure 7:**
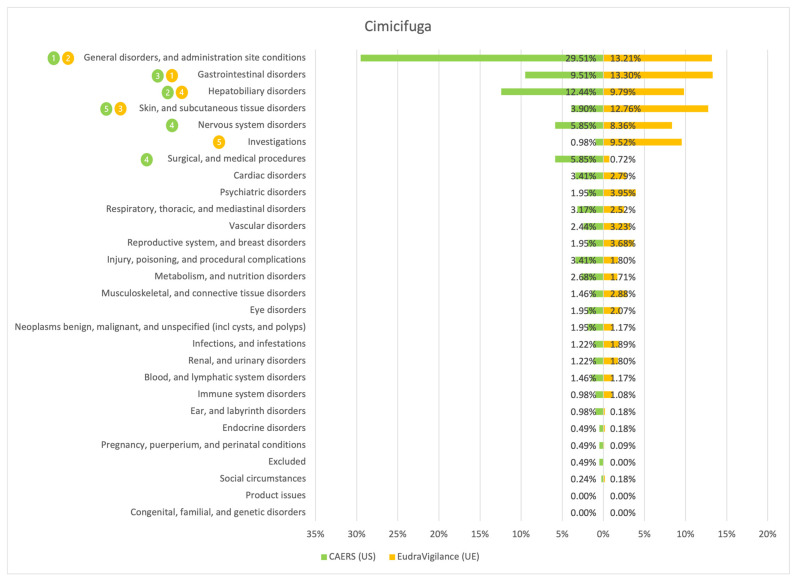
Summary of the frequency of reporting side effects of Cimicifuga in the US and EU registers. Data marked in green refer to the CAERS (US) database and yellow to the EudraVigilance (UE) database. Numbers 1–5 present the most commonly reported categories of adverse effects in descending order.

The results of the comparison between the American (CAERS) and the European (EudraVigilance) registers are presented in the tables and graphs below (Figure 1, Figure 2, Figure 3, Figure 4, Figure 5, Figure 6 and Figure 7). As far as all the graphs showing the distribution of the types of adverse effects possibly caused by each of the seven studied ingredients are concerned, the data is presented as a percentage of all cases of adverse effects attributed to the particular ingredient in the particular register. The categories were sorted in a descending order according to sums of events in a particular category in both registers. By using the 80/20 method for both registers, the five most frequently occurring categories of adverse effects were indicated in graphic form, colour-coded, i.e., green graphical and numerical symbols denote the CAERS register; yellow—EudraVigilance. According to the method used, it was assumed that the majority of notifications fall into the five most frequently appearing categories.

## 3. Results

A list of the analysed reports issued by ANSES is presented in Table 1 below. Opinions on seven borderline ingredients selected after comparison of the French Nutrivigilance and the EudraVigilance databases are marked.

Regardless of the studied region or the analysed ingredient, the five most frequently occurring disorders always included general ones and those conditions connected with the functioning of the gastrointestinal tract.

The prevalent sex in all the studied reports was female.

On the basis of the data sourced from the Food and Drug Administration (USA), in relation to the seven studied ingredients, the authors concluded that adverse effects potentially caused by them are usually classified as general disorders. Considering the frequency of symptoms, a similarity between the European and the American data was observed in six out of 35 possible cases. In the other cases, the frequency of their occurrence in both studied regions diverged.

Adverse effects were reported the most often by patients and consumers aged 18–64, apart from reports concerning preparations containing glucosamine and chondroitin.

Among the categories of symptoms reported frequently by American consumers the following can be indicated: general disorders and administration site conditions; gastrointestinal disorders; skin and subcutaneous tissue disorders; nervous system disorders; and hepatobiliary disorders.

Those patients whose notifications were analysed within the framework of the EudraVigilance system usually experienced similar categories of symptoms, apart from the fact that they mentioned “investigations” more often than hepatobiliary disorders. 

All the studied ingredients are prevalent in dietary supplements registered in Poland.

When comparing the prevalence of products containing the studied ingredients in the Polish register of functional food products and in the American database of labels, it can be observed that the number of products submitted in Poland amounts to, on average, 40% of the total number of products registered in the Dietary Supplements Labels Database.

## 4. Discussion

In all databases, among the products identified as borderline, a similarity in the distribution of adverse effects according to sex and age groups can be observed, regardless of whether they are a medicine or a dietary supplement. This finding is corroborated by the fact that marketing practices indicate a wider use of plant-based dietary supplements rather than a strict definition, were observed in previous years [37]. This indicates that from the consumer’s point of view, the effect that the consumption of a product or ingredient will have is more important than its legal status. Hence, it seems reasonable to suggest that studies concerning adverse effects taking data sourced from registers of medical products as well as dietary supplements should be conducted. Plant-based ingredients should be subjected to detailed analysis as their consumption is associated with adverse effects, especially those connected with the liver, more often than with other groups of ingredients [38]. It is worth emphasising that in this study, four out of seven analysed ingredients and plant derivatives commonly present in the dietary supplements are registered on the Polish market.

Dietary supplements that contain the analysed ingredients are present in both the American and the Polish markets, from the data of the national (Polish) register of functional foods and the American Dietary Supplements Label Database. This suggests that Polish consumers are potentially subjected to health hazards connected with the consumption of certain dietary supplements.

Importantly, it must be remembered that in order to achieve desired health effects and avoid adverse reactions one must take recommended doses of supplements and adhere to their suggested use [39]. Thus, when a particular ingredient is analysed, it is worth collecting data concerning its properties from all available sources.

Adverse effects attributed to dietary supplements are often categorised as general disorders. This phenomenon is particularly visible in the American database of reports on adverse effects. The reliability of such reports is questionable as it begs the question whether collecting data on worrying health symptoms resulting from a potential association made with the consumption of a particular dietary supplement and publishing it uncritically may not lead to unnecessary stigmatisation of the products. Although raw data may be an important source of information for independent scientific studies [40], in this context, systems based on a more in-depth scientific analysis of the registered notifications—such as the French register, which was the starting point of this study—may have a certain advantage. 

When analysing data on the adverse effects of dietary supplements polypragmasia and the compound character of some preparations are also problematic. Research shows that consumers who use numerous products (medicines and dietary supplements) concurrently more often experience adverse health effects. Similar effects may be caused by using compound dietary preparations. Multivitamin preparations are responsible for a significant proportion of the reported effects [41]. This is important, as since the COVID-19 pandemic there is a trend on the Polish market observed that universal and multidirectional dietary supplements have a growing popularity [42]. This emphasises the importance of information about all concurrently used drugs and dietary supplements which should be included during the adverse effect submission process. Consumers should have possibility to choose all products used at the same time, preferably from the drop-down list, to make this process simple and keep data consistent [43].

It is also worth mentioning that consumers and medical providers in Poland can inform the Chief Sanitary Inspector on adverse effects caused by dietary supplements, who forwards the notification for investigation to the Dietary Supplements Team. As can be concluded from the reply to our inquiry addressed to this official body, in the years 2007–2022 the Chief Sanitary Inspector has received only two such notifications, while the situations they described did not form a basis for issuing any scientific recommendations for the industry.

Monitoring of the use of supplements seems to be particularly important in those countries where no authorisation procedures are required before the product is placed on the market [44]. In most European Union countries, notification systems for dietary supplements are in place, but only a few include authorisation procedures based on quality assessment before product release [36]. Following the example of France, some EU countries implemented their own, national dietary supplements adverse effects reporting systems [45], However, many still have no procedures for monitoring or collecting dietary supplements’ adverse reactions. Examples include Romania [20] and Croatia [46]. The procedure of notification functioning in Poland, even in spite of its comprehensive character, does not always guarantee that products which do not potentially comply with regulations and are thus dangerous will be withdrawn from the market [43]. This is an argument for the need to implement a full-scale system for the collection and processing of information concerning the adverse effects caused by dietary supplement in Poland, and also in other countries with less developed food safety monitoring systems.

The Italian experience shows that systems based on spontaneous adverse effects reports can improve awareness of health professionals and patients in the matter of the safety of natural health-supporting products, especially plant-based supplements [47]. 

However, it seems to be difficult to consider dietary supplements’ adverse effects separately from drugs’ adverse effects (ADEs) because both these categories have different regulatory statuses and some dietary supplement manufacturers produce actual drug-like preparations [48]. The ADEs often identified in research include cardiovascular, gastrointestinal, and nervous system disorders [49]. Thus, it is justified to develop a dietary supplements’ adverse effects monitoring system.

On the other hand, large-scale surveys on negative health effects associated with the parallel use of dietary supplements have successfully identified ingredients with a high risk of adverse effects. In the same country (Italy), data collected by an official system was insufficient to achieve such results [50].

It is challenging to create a model of such a system that would function highly efficiently, according to its objectives. The American experiences show that determining the procedure of reporting adverse effects at the level of legislation improves the function of a register [51]. As important as determining the rules is promoting educational activities in society and conducting research on the safety of dietary supplements [52].

### Limitations

It should be emphasised that due to the method of selection of cases from the American register, i.e., based solely on the name of product, a partial overlap occurs in the area of data concerning glucosamine and chondroitin as both these ingredients commonly occur together in products dedicated to joint support. 

In the case of the European register, notifications of adverse effects of supplements are in most cases made by healthcare professionals who—when reporting—also include other medicines and preparations consumed by the patient. The described symptoms are grouped into 27 categories. Data used in this research, although often generated by healthcare professionals, cannot be used for determining the likelihood of experiencing side effects [53].

An attempt to compare adverse effects reported in Europe and the USA is thus subject to a certain risk of error—firstly, due to the unknown distribution of the reported data between professional entities and individuals; secondly, due to possible interpretative errors of symptoms described in the American register made at the stage of their grouping according to the European register’s categories. In the case of the American register, notifications can be made by any person, i.e., a medical professional, a patient, or their family. The terms that describe symptoms are often imprecise, non-medical, with a single symptom possibly denoted by several different names. It is impossible to verify the truthfulness of the provided information; it also needs to be remembered that intentions of the reporting user may be connected with the desire to obtain compensation or express their bitterness—as best illustrated by those notifications that indicate “no effect whatsoever!” Among the adverse effects, American consumers also report health conditions whose connection with consumption of dietary supplements is questionable (e.g., broken ankle, sore throat, pneumonia, or the necessity to perform diagnostic tests such as mammography or chest CT scan). Finally, reported cases are not the subject of a risk analysis by healthcare professionals [54], so the cause and effect relationship cannot be established.

Particular interpretative ambiguities of symptoms indicated in the American register occurred at reported high/low results of lab tests. In the case of parameters assigned to a specific organ or system, the reported symptom was grouped in the appropriate category, e.g., high serum creatinine to urinary tract disorders. In situations when deviation from the norm may have been the result of various conditions/disorders, e.g., high serum sodium, the authors categorised them as “investigations”. Those ‘symptoms’ that could not be regarded as symptoms (e.g., “CT scan”) and unambiguously grouped under a single category were marked as “excluded” and removed from further analysis.

## 5. Conclusions

As a result of the analyses, the following relationships were formulated:

- Considering the system of notification of dietary supplements newly placed on the market in force in Poland and its current flaws and imperfections, it is reasonable to undertake work on creating a register of adverse effects caused by dietary supplements.
- The studied ingredients are commonly present in dietary supplements placed on the Polish market and may be simultaneously available as OTC medicines or plant-based medicines. The number of notifications recorded in Poland’s national register indicates a real risk of producing the adverse effects identified in the CAERS register.
- Non-professional entities generate more general-type notifications, whose scientific and substantive value is lower, but which may nonetheless identify certain trends and needs for further, more in-depth studies.
- Owing to the multidrug approach of patients who also consume dietary supplements, as well as the role that dietary supplements play in causing adverse effects, the concept of a national register should include the possibility to report other concurrently used medicinal preparations and functional food products.

## Figures and Tables

**Table 1:** The list of ANSES’ reports taken to the research, including reports on ingredients present simultaneously in the French Nutrivigilance and EudraVigilance databases and recognised as borderline.

Date of Issue	Title of Document	Ingredients or Other Factors That Constitute a Probable Cause of the Adverse Effect
14 December 2007	Opinion of the French Food Safety Agency on the use of botanical food supplements which have been the subject of drug safety monitoring reports	Hoodia Gordoni Cimcifuga racemosa ^+^ Desmodium Viola tricolor Viola arvensis Echinacea angustifolia ^+^ Echincea pallida ^+^ Echinacea purpurea ^+^ Polygonum multiflorum
7 June 2010	Opinion of the French Food Safety Agency on the safety of use of a plant (Galega officinalis) in food supplements	Galega officinalis
22 November 2010	Opinion of the French Agency for Food, Environmental and Occupational Health and Safety regarding the safety of yam (Dioscorea) alcohol extracts in food supplements	Dioscorea
25 February 2011	Opinion of the French Agency for Food, Environmental and Occupational Health and Safety regarding the risk of drug eruption following the consumption of lutein and zeaxanthin in food supplements	Lutein Zeaxanthin
7 August 2012	ANSES OPINION regarding the safety of consumption of powdered green tea in food supplements	Green tea ^+^
14 February 2014	OPINION of the French Agency for Food, Environmental and Occupational Health and Safety on the risks associated with the presence of “red yeast rice” in food supplements	Red yeast rice
14 March 2014	OPINION of the French Agency for Food, Environmental and Occupational Health and Safety on the risks associated with the presence in food supplements of p-synephrine or ingredients obtained from Citrus spp. fruits containing this substance	p-synephrine
31 October 2014	ANSES OPINION on allergy to a food supplement containing spirulina	Spirulina
27 March 2015	ANSES OPINION on a case of hepatitis following the consumption of a food supplement for joint conditions	Glucosamine ^+^ Chondroitin ^+^
n/a	ANSES OPINION AND REPORT on the risks associated with the consumption of food supplements for sportsmen whose purpose is muscle building or adipose tissue reduction	n/a
19 April 2017	ANSES OPINION regarding photo-induced drug eruption following the consumption of a Bio-butter food supplement	Butyric acid
4 August 2017	ANSES OPINION on the risks associated with the consumption of food supplements containing spirulina	Spirulina
23 February 2018	ANSES OPINION on the risks associated with the consumption of food supplements containing melatonin	Melatonin ^+^
23 May 2018	ANSES OPINION on allergy to the food supplement Nutrilin^®^	Linseed extract
23 May 2018	ANSES OPINION on three cases of allergy to food supplements containing pollen or hive products	Hive
16 January 2019	ANSES OPINION on a case of severe hypokalaemia following misuse of the food supplement Rhubarbe^®^ containing liquorice	Liquorice ^+^ Rhubarb
1 August 2019	ANSES OPINION on the “safety of use of berberine-containing plants in the composition of food supplements”	Berberine
16 October 2019	ANSES OPINION on a case of severe hypokalaemia following misuse of an oral food supplement	Consumer’s error
24 December 2019	ANSES OPINION regarding anaphylaxis associated with consumption of a food supplement containing passiflora	Passiflora
1 April 2020	ANSES OPINION on a case of fatal fulminant hepatitis associated with consumption of the food supplement Slim Metabol^®^	Garcinia cambogia
19 June 2020	ANSES OPINION on two cases of severe acute life-threatening hepatitis associated with consumption of the food supplement Chewable Hair Vitamins^®^	Ingredient not determined
26 January 2021	ANSES OPINION regarding oesophageal perforation associated with the consumption of the food supplement Préservion 3 capsules	Large capsule
28 July 2021	ANSES OPINION regarding vitamin D poisoning in infants following misuse of food supplements	Vitamin D
4 October 2021	ANSES OPINION regarding epileptic seizures associated with consumption of the food supplement Novanuit^®^ Triple Action	Melatonin ^+^

+ sign indicates ingredients present simultaneously in the French Nutrivigilance and EudraVigilance databases and recognised as borderline.

**Table 2:** Comparison of data on Melatonin from different databases.

	US	UE	PL
KEY WORD	Melatonin	Melatonin	Melatonin *
NUMBER OF REGISTERED DS	1696		786
NUMBER OF CASES	463	2658	
AGE GROUP			
Not Specified	13.6%	22.2%	
0–1 Month		0.3%	
2 Months–2 Years	0.4%	0.6%	
3–11 Years	4.1%	10.5%	
12–17 Years	2.4%	9.2%	
18–64 Years	51.2%	37.7%	
65–85 Years	25.9%	16.3%	
More than 85 Years	2.4%	3.2%	
SEX			
Female	67.6%	57.8%	
Male	28.5%	38.3%	
Not Specified	3.9%	3.8%	
REPORTER GROUP			
Healthcare Professional		65.2%	
Non-Healthcare Professional		34.7%	
Not Specified	100%	0.1%	

Star sign (*) indicates any string of characters.

**Table 3:** Comparison of data on Liquorice from different databases.

Key Word	US Liquorice	UE Glycyrrhiza Glabra L. Radix	PL Lukrecj *
NUMBER OF REGISTERED DS	3091		1191
NUMBER OF CASES	27	234	
AGE GROUP			
Not Specified	22.2%	2.1%	
0–1 Month			
2 Months–2 Years		0.9%	
3–11 Years	3.7%	1.7%	
12–17 Years		1.3%	
18–64 Years	44.4%	55.1%	
65–85 Years	29.6%	32.9%	
More than 85 Years		6.0%	
SEX			
Female	70.4%	56.4%	
Male	25.9%	42.7%	
Not Specified	3.7%	0.9%	
REPORTER GROUP			
Healthcare Professional		93.6%	
Non-Healthcare Professional		5.6%	
Not Specified	100%	0.9%	

Star sign (*) indicates any string of characters.

**Table 4:** Comparison of data on Green Tea from different databases.

	US	UE	PL
Key Word	Green Tea	Camellia Sinensis *	Zielon * + Herbat *
NUMBER OF REGISTERED DS	6259		3595
NUMBER OF CASES	223	171	
AGE GROUP			
Not Specified	24.7%	21.6%	
0–1 Month			
2 Months–2 Years	1.9%		
3–11 Years			
12–17 Years	0.9%	0.6%	
18–64 Years	55.6%	70.2%	
65–85 Years	18.4%	7.6%	
More than 85 Years	0.4%		
SEX			
Female	65.9%	60.8%	
Male	31.8%	35.7%	
Not Specified	2.2%	3.5%	
REPORTER GROUP			
Healthcare Professional		69.6%	
Non-Healthcare Professional		29.8%	
Not Specified	100%	0.6%	

Star sign (*) means any string of characters.

**Table 5:** Comparison of data on Glucosamine from different databases.

	US	UE	PL
Key Word	Glucosamine *	Glucosamine *	Glukozamin *
NUMBER OF REGISTERED DS	3221		1408
NUMBER OF CASES	673	1543	
AGE GROUP			
Not Specified	14.7%	16.3%	
0–1 Month		0.1%	
2 Months–2 Years		0.4%	
3–11 Years		0.6%	
12–17 Years		0.6%	
18–64 Years	33.9%	33.1%	
65–85 Years	44.9%	45.4%	
More than 85 Years	6.5%	3.5%	
SEX			
Female	64.2%	70.8%	
Male	34.2%	26.9%	
Not Specified	1.6%	2.3%	
REPORTER GROUP			
Healthcare Professional		72.10%	
Non-Healthcare Professional		27.50%	
Not Specified	100%	0.40%	

Star sign (*) indicates any string of characters.

**Table 6:** Comparison of data on Echinacea from different databases.

	US	UE	PL
Key Word	Echinacea *	Echinacea *	Jeżówk *
NUMBER OF REGISTERED DS	2901		498
NUMBER OF CASES	82	347	
AGE GROUP			
Not Specified	15.9%	15.9%	
0–1 Month		0.3%	
2 Months–2 Years	1.2%	3.7%	
3–11 Years	3.7%	4.9%	
12–17 Years		2.0%	
18–64 Years	43.9%	57.6%	
65–85 Years	32.9%	14.7%	
More than 85 Years	2.4%	0.9%	
SEX			
Female	65.9%	70.0%	
Male	34.1%	27.4%	
Not Specified		2.6%	
REPORTER GROUP			
Healthcare Professional		68.6%	
Non-Healthcare Professional		29.1%	
Not Specified	100%	2.3%	

Star sign (*) indicates any string of characters.

**Table 7:** Comparison of data on Chondroitin from different databases.

	US	UE	PL
Key Word	Chondroitin	Chondroitin *	Chondroityn *
NUMBER OF REGISTERED DS	1713		1120
NUMBER OF CASES	362	758	
AGE GROUP			
Not Specified	14.4%	20.1%	
0–1 Month			
2 Months–2 Years		0.4%	
3–11 Years		0.4%	
12–17 Years		0.7%	
18–64 Years	32.0%	35.4%	
65–85 Years	46.1%	39.1%	
More than 85 Years	7.5%	4.1%	
SEX			
Female	68.8%	66.6%	
Male	37.6%	27.6%	
Not Specified	1.7%	5.8%	
REPORTER GROUP			
Healthcare Professional		62.3%	
Non-Healthcare Professional		36.8%	
Not Specified	100%	0.9%	

Star sign (*) indicates any string of characters.

**Table 8:** Comparison of data on Cimicifuga from different databases.

	US	UE	PL
Key Word	Black Cohosh	Cimicifuga *	Pluskwic *
NUMBER OF REGISTERED DS	947		96
NUMBER OF CASES	85	558	
AGE GROUP			
Not Specified	20.0%	13.6%	
0–1 Month		0.2%	
2 Months–2 Years			
3–11 Years			
12–17 Years		0.4%	
18–64 Years	67.1%	79.4%	
65–85 Years	12.9%	6.3%	
More than 85 Years		0.2%	
SEX			
Female	95.3%	96.8%	
Male		1.8%	
Not Specified	4.7%	1.4%	
REPORTER GROUP			
Healthcare Professional		66.7%	
Non-Healthcare Professional		32.4%	
Not Specified	100%	0.9%	

Star sign (*) indicates any string of characters.

## Data Availability

Publicly available datasets were analysed in this study. This data can be found at: https://powiadomienia.gis.gov.pl (accessed on 10 October 2022); https://www.anses.fr/en/content/anses-request-based-opinions-and-reports (accessed on 4 July 2022); https://www.fda.gov/food/compliance-enforcement-food/cfsan-adverse-event-reporting-system-caers#files (accessed on 29 July 2022); https://dap.ema.europa.eu/analytics/saw.dll?PortalPages&PortalPath=%2Fshared%2FPHV%20DAP%2F_portal%2FDAP&Action=Navigate&P0=1&P1=eq&P2=%22Line%20Listing%20Objects%22.%22Substance%20High%20Level%20Code%22&P3=1+25657 (accessed 8 August 2022); https://dap.ema.europa.eu/analytics/saw.dll?PortalPages&PortalPath=%2Fshared%2FPHV%20DAP%2F_portal%2FDAP&Action=Navigate&P0=1&P1=eq&P2=%22Line%20Listing%20Objects%22.%22Substance%20High%20Level%20Code%22&P3=1+23281 (accessed 8 August 2022); https://dap.ema.europa.eu/analytics/saw.dll?PortalPages&PortalPath=%2Fshared%2FPHV%20DAP%2F_portal%2FDAP&Action=Navigate&P0=1&P1=eq&P2=%22Line%20Listing%20Objects%22.%22Substance%20High%20Level%20Code%22&P3=1+58751 (accessed 8 August 2022); https://dap.ema.europa.eu/analytics/saw.dll?PortalPages&PortalPath=%2Fshared%2FPHV%20DAP%2F_portal%2FDAP&Action=Navigate&P0=1&P1=eq&P2=%22Line%20Listing%20Objects%22.%22Substance%20High%20Level%20Code%22&P3=1+19434 (accessed 8 August 2022); https://dap.ema.europa.eu/analytics/saw.dll?PortalPages&PortalPath=%2Fshared%2FPHV%20DAP%2F_portal%2FDAP&Action=Navigate&P0=1&P1=eq&P2=%22Line%20Listing%20Objects%22.%22Substance%20High%20Level%20Code%22&P3=1+30122 (accessed 8 August 2022); https://dap.ema.europa.eu/analytics/saw.dll?PortalPages&PortalPath=%2Fshared%2FPHV%20DAP%2F_portal%2FDAP&Action=Navigate&P0=1&P1=eq&P2=%22Line%20Listing%20Objects%22.%22Substance%20High%20Level%20Code%22&P3=1+24818 (accessed 8 August 2022); https://dap.ema.europa.eu/analytics/saw.dll?PortalPages&PortalPath=%2Fshared%2FPHV%20DAP%2F_portal%2FDAP&Action=Navigate&P0=1&P1=eq&P2=%22Line%20Listing%20Objects%22.%22Substance%20High%20Level%20Code%22&P3=1+169371 (accessed 8 August 2022).
